# Plant diversity has contrasting effects on herbivore and parasitoid abundance in *Centaurea jacea* flower heads

**DOI:** 10.1002/ece3.3142

**Published:** 2017-10-05

**Authors:** Norma Nitschke, Eric Allan, Helmut Zwölfer, Lysett Wagner, Sylvia Creutzburg, Hannes Baur, Stefan Schmidt, Wolfgang W. Weisser

**Affiliations:** ^1^ Institute of Ecology Friedrich‐Schiller‐University Jena Germany; ^2^ Institute of Plant Sciences University of Bern Bern Switzerland; ^3^ Department for Animal Ecology I University of Bayreuth Bayreuth Germany; ^4^ Abteilung Wirbellose Tiere Naturhistorisches Museum Bern Bern Switzerland; ^5^ Institute of Ecology and Evolution University of Bern Bern Switzerland; ^6^ Bavarian State Collection of Zoology (ZSM) Munich Germany; ^7^ Present address: Terrestrial Ecology Research Group Department of Ecology and Ecosystem Management School of Life Sciences Weihenstephan Technical University of Munich Freising Germany

**Keywords:** chalcid wasps, grassland, *Jena Experiment*, Tephritidae, tri‐trophic system

## Abstract

High biodiversity is known to increase many ecosystem functions, but studies investigating biodiversity effects have more rarely looked at multi‐trophic interactions. We studied a tri‐trophic system composed of *Centaurea jacea* (brown knapweed), its flower head‐infesting tephritid fruit flies and their hymenopteran parasitoids, in a grassland biodiversity experiment. We aimed to disentangle the importance of direct effects of plant diversity (through changes in apparency and resource availability) from indirect effects (mediated by host plant quality and performance). To do this, we compared insect communities in *C. jacea* transplants, whose growth was influenced by the surrounding plant communities (and where direct and indirect effects can occur), with potted *C. jacea* plants, which do not compete with the surrounding plant community (and where only direct effects are possible). Tephritid infestation rate and insect load, mainly of the dominant species *Chaetorellia jaceae*, decreased with increasing plant species and functional group richness. These effects were not seen in the potted plants and are therefore likely to be mediated by changes in host plant performance and quality. Parasitism rates, mainly of the abundant chalcid wasps *Eurytoma compressa* and *Pteromalus albipennis*, increased with plant species or functional group richness in both transplants and potted plants, suggesting that direct effects of plant diversity are most important. The differential effects in transplants and potted plants emphasize the importance of plant‐mediated direct and indirect effects for trophic interactions at the community level. The findings also show how plant–plant interactions critically affect results obtained using transplants. More generally, our results indicate that plant biodiversity affects the abundance of higher trophic levels through a variety of different mechanisms.

## 1 INTRODUCTION

Several studies have shown that plant diversity affects the diversity and abundance of other trophic levels (e.g., Scherber et al., 2010). However, the mechanisms driving these effects remain unclear because impacts of plant diversity on trophic interactions, such as parasitism or predation, are only rarely investigated (Ebeling, Klein, Weisser, & Tscharntke, 2012). Pest control by natural enemies has been studied in agro‐ecological research (e.g., Bianchi, Booij, & Tscharntke, 2006; Menalled, Marino, Gage, & Landis, 1999; Thies & Tscharntke, 1999) where higher parasitoid efficiency, that is, higher parasitism rates of insect herbivores, is often found in more structurally complex or species‐rich systems (Andow, 1991; Langellotto & Denno, 2004; Price et al., 1980). This is often referred to as the *Enemies Hypothesis* (Root, 1973). However, most of these studies addressed isolated trophic levels or did not consider effects of plant diversity. The few studies that did examine plant diversity effects on parasitism used plant communities with very few species, that is, a maximum diversity of three species. More recently, multi‐trophic interactions have been studied in experimentally manipulated plant communities with longer plant diversity gradients, where plant diversity effects were shown to cascade up the food chain (e.g., Ebeling et al., 2012, 2014; Petermann, Müller, Weigelt, Weisser, & Schmid, 2010). However, these studies were not able to look in detail at the mechanisms driving these cascading diversity effects.

Bottom‐up effects of biodiversity on trophic interactions may be caused by both direct effects of the plant community and indirect effects mediated by changes to host plant performance. Plant diversity can directly affect higher trophic levels by increasing the complexity of the olfactory, optical, and structural environment. This may mask the odors or visual cues that herbivores use to find their host plants (Coll & Bottrell, 1994; Finch & Collier, 2000; Randlkofer, Obermaier, Hilker, & Meiners, 2010) making the hosts less apparent to the herbivore. Particular plant species may also cause this effect through associational resistance (Barbosa et al., 2009): for example, similarly colored neighboring plants may attract insects away from the host plant, or volatiles emitted by neighboring plants may repel herbivores or reduce their ability to find the host plant. Diversity effects may be driven by a number of such neighboring species and therefore by a general increase in complexity in species‐rich communities. A lack of alternative hosts in the plant community, as may occur in (diverse) communities of taxonomically distant plant species, may also reduce food supply for herbivores and therefore herbivory rates on particular target plants (Jactel & Brockerhoff, 2007; Root, 1973).

As well as reducing herbivore abundance, high plant diversity could also reduce the efficiency of parasitoids in finding their insect hosts by increasing structural complexity and producing odor blends that mask the focal plant (Andow & Prokrym, 1990; Bukovinszky, Gols, Hemerik, van Lenteren, & Vet, 2007; Gols et al., 2005; Randlkofer, Obermaier, & Meiners, 2007). Alternatively, predators and parasitoids could benefit from plant diversity if diverse plant communities provide a greater range of food sources, such as more floral resources (nectar and pollen) for parasitoids (Araj, Wratten, Lister, & Buckley, 2008; Lavandero, Wratten, Didham, & Gurr, 2006) or a greater diversity of prey for generalist predators (Root, 1973). Parasitoids could also operate more efficiently when herbivore abundance is low (Ebeling et al., 2012), as would be expected in diverse plant communities. However, lower herbivore abundance may also decrease parasitism rates (e.g., White & Andow, 2005) if patch tenure time is longer in high‐density patches, as predicted by several patch time allocation models (Van Alphen & Bernstein, 2008). Analyses controlling for herbivore abundance are necessary to test these ideas.

In addition to these direct effects of the plant community on higher trophic levels, plant diversity can also indirectly affect herbivore and predator communities. Indirect effects arise when plant diversity influences the growth and nutrient levels of host plants (e.g., Nitschke et al., 2010; Roscher, Kutsch, & Schulze, 2011), which in turn affects insect herbivores (Awmack & Leather, 2002; Mattson, 1980). We consider these effects to be more indirect than effects of plant diversity mediated by structural, odor, or resource diversity because they are caused by an effect of the plant community on individual plants, mediated by changes in competition, which in turn affects higher trophic levels. In contrast, changes in structural, odor, or resource diversity occur as a direct consequence of changed plant community diversity. A reduction in individual plant performance with increasing diversity is likely to reduce the availability of resources for herbivores in general, such as by reducing the number of flower heads for flower feeding herbivores. The performance of individual plants might be reduced if they suffer more competition: for instance, plant tissue nutrient levels may be reduced in diverse communities due to more efficient nutrient use in species‐rich assemblages (van Ruijven & Berendse, 2005) and/or due to increased light competition, which causes plants to invest more in structural, carbon‐rich tissues (Hirose & Werger, 1995). The lower plant quality could reduce the abundance or performance of herbivores in diverse plant communities. Complex effects may also occur; for example, Kigathi, Weisser, Veit, Gershenzon, and Unsicker (2013) showed that plants may change their emission of volatile compounds when growing in competition with other plants, which has effects on the attraction of herbivores and their natural enemies. Overall, effects on the third trophic level are likely to be weaker as they are even more indirect (Kagata, Nakamura, & Ohgushi, 2005; Scherber et al., 2010).

To separate these direct and indirect effects, we used transplanted and potted host plants (the common knapweed, *Centaurea jacea*) placed into experimental plant communities differing in species richness. We then analyzed the response of its flower head infesting Tephritidae and the hymenopteran parasitoids attacking those Tephritidae to the diversity of the plant community. Host plants that were transplanted into experimental plant communities (transplants) interacted with the surrounding plant community over a number of years, both aboveground (mainly light competition) and belowground (root/nutrient competition). Plant diversity can have both direct and indirect effects on the herbivore and parasitoid community of these transplants. We compared responses on these transplants with potted plants that were placed inside the experimental plant communities during the study period only (potted plants) and which did not interact belowground with the surrounding plant community. Aboveground, there may have been limited interaction (short period of potential light competition) with other plants in the community. In this case, plant diversity can have only direct effects on higher trophic levels because indirect effects mediated by changes in plant performance or quality are excluded. Other aboveground interactions between plants, such as communication via volatile organic compounds (VOCs) could occur in both host plant types; however, we did not investigate them as a source of diversity effects as this would require separate experiments.

We hypothesize that herbivore abundance depends both on indirect (host plant quality/performance) and direct effects (community characteristics) of plant diversity. We therefore expect to find that plant diversity reduces herbivore abundance in both types of host plants although we might anticipate stronger effects in transplants where both direct and indirect effects operate. To test for the mechanisms driving potential direct and indirect effects, we can model the effect of other plant community parameters on the herbivores. If resource density effects are important, then we would expect that the presence of alternative hosts in the community increased herbivory. If apparency drives the effects, then a measure of apparency, such as the height of host plants relative to the rest of the community (which indicates how easy it is for insects to find their host using visual cues), should significantly increase herbivory. Structural heterogeneity might reduce herbivory, which means that herbivory is lower when LAI, which can serve as a proxy for structural complexity, is high. To test for indirect effects mediated by host plant performance, we can include a measure of resource density per host plant. For parasitoids, we hypothesize indirect effects to be of minor importance and expect positive direct effects of plant diversity. We should therefore see similar effects of plant diversity on parasitoids in both transplants and potted plants. Parasitoids are likely to respond strongly to structural complexity (strong effects of LAI would be hypothesized in this case) and to resource density (quantity of tephritid hosts). We test for these effects using a large grassland diversity experiment, the *Jena Experiment* (Roscher et al., 2004). The *Jena Experiment* contains experimental plant communities of 20 × 20 m^2^, which differ in plant species richness and the number of plant functional groups. We analyzed data from experimental plant communities containing 1–8 species and 1–4 plant functional groups (i.e., grasses, legumes, small herbs, tall herbs).

## 2 MATERIALS AND METHODS

### 2.1 The study system

*Centaurea jacea* L. s. l. (brown knapweed; Asteraceae) is native to Eurasia and common throughout Germany. The long‐lived perennial hemicryptophyte (plants with overwintering buds at soil level) re‐emerges in spring (Press & Gibbons, 1993) producing vegetative side rosettes, flowers, and fruits between June and October (Jongejans, de Kroon, & Berendse, 2006). *C. jacea* flower heads are widely attacked by Tephritidae, an abundant family of Diptera that mainly inhabit fruits or other seed‐bearing organs of flowering plants (White, 1988). Six species of Tephritidae, with flight periods between May and September, are associated with *C. jacea* in Germany. Four of them have a narrow host range (i.e., either monophagous on *C. jacea* or using a few *Centaurea* species only (Merz, 1994; White, 1988): *Acinia corniculata* (Zetterstedt), *Urophora quadrifasciata* (Meigen), *Urophora jaceana* (Hering), and *Chaetorellia jaceae* (Robineau‐Desvoidy), the latter two are likely to be monophagous on *C. jacea* in the study area (HZ, pers. obs.). Two species are associated with more than 15 composite host plant species of different genera (Merz, 1994): *Acanthiophilus helianthi* (Rossi), *Chaetostomella cylindrica* (Robineau‐Desvoidy). These Tephritidae have different foraging behaviors ranging from destructive feeding on the flower head (*C. jaceae*) to inducing complex woody galls in the capitulum (*U. jaceana*); however, detailed information is not available for all potentially occurring species. Parasitoids of the families Eurytomidae, Pteromalidae, Eulophidae (all Chalcidoidea), Braconidae, and Ichneumonidae attack these flower head phytophages in great numbers (Dempster, Atkinson, & Cheesman, 1995; Varley, 1947; Zwölfer, 1988). For tephritid hosts, chalcid wasps are the major parasitoids and these have a broad host range (see Figure S1 for a potential interaction web based on Tephritidae attacking *C. jacea*). Since the study site is mown twice a year, Tephritidae and their parasitoids, which commonly overwinter in flower heads of the host plant, recolonize the experimental field site every year from source populations in the surrounding meadows.

### 2.2 Experimental design

In order to investigate responses of the second (Tephritidae) and third trophic levels (parasitoids) to plant diversity, the study was conducted in 76 unique experimental plant communities representing a gradient of plant species (1–16) and plant functional richness levels (1–4). On plots of 20 × 20 m, set in the floodplain of the river Saale (Jena, Thuringia, Germany), plant communities were established in 2002 from a pool of 60 grassland species, representing the surrounding Arrhenatherion community. The grassland species were assigned to four plant functional groups (legumes, grasses, tall herbs, and small herbs) and mixtures of 1, 2, 4, 8, and 16 species were created by randomly selecting species from the pool of 60. Each plant species richness level was replicated on 16 plots, except for the 16‐species communities (14 plots); additionally, four plots were sown with all 60 species (for details, see Roscher et al., 2004; *The Jena Experiment* and Table S1). The design also manipulated functional group richness to be as orthogonal as possible to plant species richness: that is, there are 8‐ or 16‐species plots with only one or two functional groups present. Experimental plots were arranged in four blocks, mostly to account for the change in soil conditions with increasing distance to the river (Roscher et al., 2004). The site is managed as a typical hay meadow with vegetation cut and removed twice a year (beginnings of June and September). The diversity gradient is maintained by regular weeding. For the current experiment, two monocultures with low target plant cover were omitted. Description of the insect community is based on collections across a gradient of 1–16 plant species, whereas analyses of insect responses are based on collections across a gradient of 1–8 plant species (see below).

We used two types of *C. jacea* host plants to study tephritid infestation and parasitism rates. Transplants were planted into the experimental plots in 2005 and as they interacted with the surrounding plant community, both direct and indirect effects of plant diversity can be expected. Additionally, we used potted plants that were placed into the experimental plots in the study year 2008. These plants did not interact at all with the surrounding plant communities belowground and would have experienced only minimal aboveground competition. Only direct plant diversity effects can be expected in the potted plants. Aboveground interactions between plants via VOCs can occur in both plant types.

Transplants were grown from seed (supplier: Rieger‐Hofmann GmbH, Blaufelden‐Raboldshausen, Germany) and planted into experimental plots 3 years after establishment of the experiment (i.e., in 2005). Five plants were planted in each plot, arranged in a row and spaced 25 cm apart. Transplants were regularly mown, along with the rest of the plant community. We found that their survival strongly decreased with increasing plant species richness (and partly with functional group richness), to the extent that none of the transplants survived in 60‐species plots and only about 30% survived in the 16‐species plots (see Nitschke et al., 2010 and Figure S4). Since plant species richness also decreases transplant biomass and the number of flower heads on plants (Nitschke et al., 2010, Figure S4), the proportion of surviving plants with flower heads was even lower (<20% in 2007, <3% in 2008, Table S2) in the 16‐species plots. We therefore excluded these communities from the analysis and sampled transplants only in plots with 1–8 plant species. Harvest took place in August (week 35) 2007 and 2008, shortly before mowing of the field site (week 36). We recorded the total number of flower heads (flowering and buds) per plant together with its height. In 2007, the flower heads (including those not yet flowering) were cut and all the flower heads of a given plant were stored together at room temperature until insects emerged; at this point, all emerging insects were identified. We then dissected 10% of the flowering flower heads per plant (i.e., those at the most advanced phenological stage), but we always dissected at least five flower heads, which could be more than 10% on plants with few flower heads. Both, unemerged insects and empty pupae of emerged insects are detected by dissection and give a clear measure of the number of infestations that occurred per flower head (*insect load*). Moreover, dissection of the single flower heads allowed for a precise determination of the proportion of flower heads that were infested (*tephritid infestation rate*). These two tephritid responses are not easily derived from pure emergence data on the plant level. However, the parasitoid community is well represented by the emerged insects, and as we assume that there are no differences in emergence success between hosts and parasitoids, we defined the *parasitism rate* to be the proportion of emerged insects that were parasitoids.

In 2008, we streamlined and standardized our data collection by only collecting 10% (and at least five) of the flower heads of each transplant (those at the most advanced phenological stage) and by storing flower heads individually at room temperature until insects emerged and could be identified. We then dissected all flower heads and additionally recorded the insects which did not emerge.

Potted plants came from a monoculture plot (3.5 × 3.5 m) established in 2002 on the same field site. Plants were dug up in mid‐May 2008 and transferred to 3‐liter pots (three plants per pot) filled with subsoil from the field site. After fertilizing once (COMPO Blaukorn, 14% N, 7% P_2_O_5_, 17% K_2_O_2_, 0.02% B, 9% S, 2% MgO), bark mulch was applied to reduce water loss from evaporation. In mid‐June (week 25), all potted plants were cut 3 cm above ground, at the same time as the experimental plots were mown. Potted plants were placed in the experimental plant communities at the beginning of July 2008 (week 28) and were watered when required. Three pots were placed on each plot and we attempted to insure the same size distribution of plants on each plot. Plants were divided into three size classes based on their number of flower heads (0–2, 3–5, and ≥6 flower heads per pot) and we placed one plant from each size class into each plot. Pots were arranged in a row and spaced 25 cm apart. We chose to add three pots per plot in order to match the number of transplants still surviving on the plots: the initial five plants per plot in 2005 had decreased to about three on average by 2008. Prior to set‐up in the plots, any flower heads that had regrown since cutting were removed from potted plants to exclude any previous flower head‐infestation (see Table S2). Potted plants had therefore grown unaffected by the communities into which they were placed. Potted plants remained in the experimental plant communities for 7 weeks, before being harvested. Aboveground interactions (e.g., shading by neighboring plants) over the course of one growing season are expected to be of minor importance for potted plant performance, compared to the impact these interactions had on the transplants over a number of years (Nitschke et al., 2010). At harvest, we recorded the total number of flower heads (flowering and bud) per pot, and their maximum height in the field. All flower heads of a pot (up to a maximum of 20) were collected and stored individually at room temperature until insects emerged. We then dissected the flower heads to identify tephritids and parasitoids that had not emerged.

We used data from separately stored flower heads to derive trophic relationships between the different species of Tephritidae and parasitoids (for details on identification and assignment of parasitoids to host species, see supplement, p.1).

### 2.3 Response variables for the higher trophic levels

We calculated a series of variables from our dissection and emergence data. We calculated *tephritid infestation rate* as the proportion of dissected flower heads that were infested, and *insect load* as the number of infestations per dissected flower head. The total number of infestations was the sum of tephritid and parasitoid individuals (because each parasitoid must have emerged from a tephritid), as well as pupae. In 2007, *parasitism rate* was defined as the proportion of all emerged insects that were parasitoids, while in 2008, *parasitism rate* was defined as the proportion of hosts that were found to be parasitized following flower head dissection. Except for three cases, parasitoids were all solitary and could be unequivocally related to tephritid hosts. In order to compare the potted plants with the transplants, we excluded the potted plant data from the 16‐species plots and analyzed all responses across the 1–8 plant species gradient for all datasets (transplants 2007 and 2008, potted 2008).

### 2.4 Variables mediating diversity and plant functional group effects on higher trophic levels

In order to identify potential mediators of diversity effects, we used a series of variables as covariates in our statistical models. *LAI* (Leaf Area Index) is a measure of above ground space use and light penetration (Welles & Norman, 1991) and commonly increases with plant species richness in experimental plant communities (Spehn, Joshi, Schmid, Diemer, & Korner, 2000). *LAI* is derived from measurements of “all light blocking objects” in a community and reflects structural complexity (Rutten, Ensslin, Hemp, & Fischer, 2015); therefore, we used this parameter as proxy for habitat complexity. Since host plant apparency (the probability that a host plant is found by its herbivore (Endara & Coley, 2011)) can affect insect herbivory (e.g., Castagneyrol, Giffard, Pere, & Jactel, 2013), we included the variable *relative height* of the host plant as an apparency measure. Relative height indicates how easy it is for insects to find their host plant using visual cues, as a plant which is much taller than its neighbors will be easy to find, whereas one that is shorter will be hard to locate. There is some evidence that Tephritidae, especially those with a narrow host range, use visual cues (especially shapes, but also size and to a lesser extent color) to find their hosts. In tests, female tephritids tended to prefer oviposition site models substantially larger than natural sites (e.g., fruits). Specifically, females of one species of *Urophora* and *Chaetorellia* (two of the genera in our samples) were shown to be most attracted toward sophisticated visual mimics of floral buds of their host plant (Díaz‐Fleischer, Papaj, Prokopy, Norrbom, & Aluja, 1999). Here, we focus on the visual apparency of the host plants as we were able to measure this. Other types of apparency, such as chemical odor apparency could also play a role but could not be quantified here. Plant community *LAI* and *height* were measured on all plots twice a year during peak standing biomass. *LAI* was recorded with an LAI‐2000 plant canopy analyzer (LI‐COR) using high resolution and a view cap masking 45° of the azimuth towards the operator. Along a 10 m transect, 10 measures were taken in the plant community (at 5 cm above ground) and combined into one mean value per plot (Weigelt et al., 2010). Floral and vegetative heights were measured every meter along a 10 m transect and averaged (Weigelt et al., 2010). Here, we used measures taken in late August 2007 and 2008. The variable *relative height* per focal plant individual (or per group of plants in a pot) was calculated by dividing *C. jacea* maximum height in the field by the larger value of the two height measures (vegetative or floral) of the respective plot. To account for resource concentration effects, we included *tephritid infestation rate* as a measure of resource density for the parasitoids. As some of the Tephritidae feed on several Asteraceae species, we also included the *presence of Asteraceae* in the communities in our models to account for potential spill overs from related hosts. To account for potential indirect effects of diversity, mediated by host plant performance, we included the *number of flower heads* per host plant as a measure of resource density for Tephritidae.

### 2.5 Statistical analysis

All analyses were conducted using the R statistical software (R Development Core Team 2010, Version 2.12.1). We tested for effects of plant diversity and plant functional composition on (i) the second (tephritids) and (ii) the third trophic level (parasitoids) using mixed effects models fitted with the “lme4” package (Bates & Sarkar, 2007). Response variables were (i) *tephritid infestation rate* and *insect load* and (ii) *parasitism rate*. In order to disentangle the direct and indirect effects of plant diversity, we analyzed each response variable for both transplants (direct and indirect effects) and potted plants (direct only). Due to low transplant survival in the 16‐species plots (Nitschke et al., 2010), we excluded them from the analysis of the transplants and, for comparability, from the analysis of the potted plants. We used three different datasets: *2007 transplants (1–8)*,* 2008 transplants (1–8)*, and *2008 potted plants (1–8)* (see Table S3 for the number of plots in each dataset).

For each of the response variables and datasets, we fitted a full model with plot as a random factor (to account for the nested design of our study, with several measures from each plot). We then carried out a two‐step analysis. In the first step, we tested for bottom‐up effects of plant diversity and functional composition and included fixed effects for *plant species richness* (log transformed), *functional group richness*, and the *presence of particular functional group*s (legumes, grasses, and small and tall herbs). As it was not possible to fit the presence of all four functional groups, together with the number of functional groups, we determined which variables should be included for each response. For each response, we fitted five models with each of the five functional group variables and excluded the variable whose model had the largest AIC value (indicated in Table 1). To account for spatial variation in tephritid and parasitoid communities, we also included block as a fixed effect (we treated it as fixed because it has only four levels and estimating variance for variables with few levels is unreliable). The full model in R syntax is as follows (shown here for the case where models with grass presence had the highest AIC):

$$\begin{array}{cc} & y\;∼\;\text{block}\;+\;\text{species richness}\;+\;\text{number of functional groups}\\ & \;\;\;+\;\text{legumes}\;+\;\text{tall herbs}\;+\;\text{small herbs}\;+\;(1|\text{plot ID}) \end{array}$$

**Table 1:**
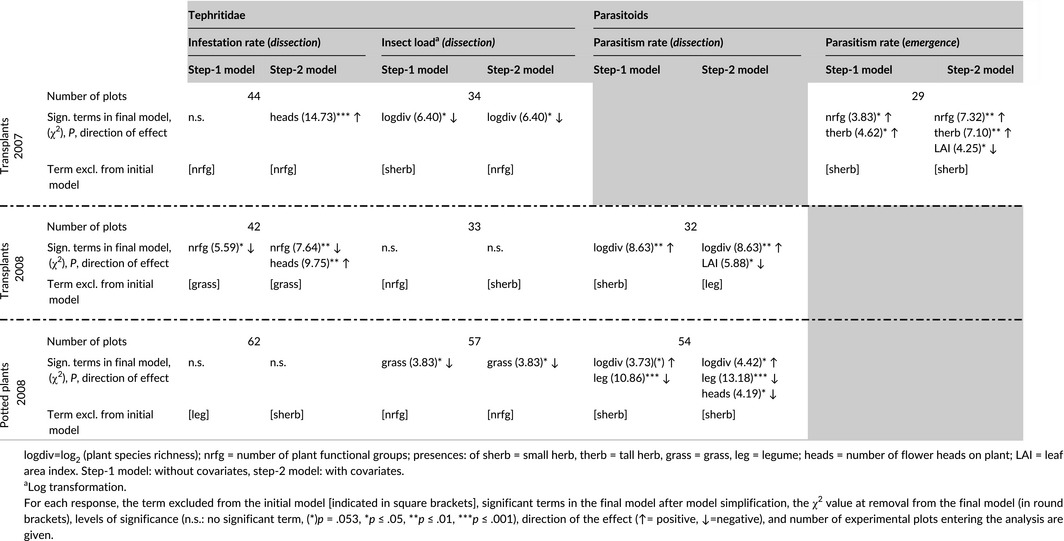
Effects of community and *Centaurea jacea* characteristics on tephritid and parasitoid responses

In a second step, we included several covariates to determine whether these mediated the effect of any of the design variables. As potential mediators of diversity effects (species richness or functional diversity), we included *community LAI* (a proxy for structural complexity), the *relative maximum height* of transplants/potted plants (a simplified measure of *C. jacea* apparency; i.e., the likelihood of a host plant being found by its herbivore) and the *number of flower heads* per host plant in the analysis of tephritid infestation, and *tephritid infestation rate* in the analysis of parasitism rate (as measure of resource density). As mediators of functional group presence (tall and small herbs), we included the *presence of Asteraceae* in the community to account for potential spill overs from related plant species. Both the tephritid species and their associated parasitoids can also use other host plants within the Asteraceae (with almost no associations occurring with plants of other families). The full model from the second step is as follows (again assuming grass presence was dropped):

$$\begin{array}{cc} & y\;∼\;\text{block}\;+\;\text{LAI}\;+\;\text{relative height}\;+\;\text{number of flower heads}\\ & \;\;\;+\;\text{Asteraceae presence}\;+\;\text{species richness}\;+\;\\ & \;\;\;\text{number of functional groups}\;+\;\text{legumes}\;+\;\\ & \;\;\;\text{tall herbs}\;+\;\text{small herbs}\;+\;(1|\text{plot ID}) \end{array}$$

In all cases, full models were simplified by progressively removing non‐significant terms, and comparing models with likelihood ratio tests to produce a minimal adequate model (final model). Significance of terms in the final model was assessed by separately removing terms from that model; however, block was always retained in the models. For *infestation rate* and *parasitism rate* (binary responses), we used a generalized linear mixed model with binomial error distributions. Other response variables were transformed if necessary in order to meet the assumption of the model (indicated in Table 1).

Figures were created in R using the packages “effects” (Fox, 2003), “plotrix” (Lemon, 2006), and “gplots” (Warnes, 2010). Data for figures were derived from the statistical model using the package “effects” (version 3.1‐2). Values for responses that were significantly affected by the predictor to be shown in the figure came from the final step‐2 model and were therefore corrected for the random effects and any other significant fixed effects. Where the response was not significantly affected by the predictor to be shown in the figure, we could not use values from a minimal adequate model and therefore took them instead from a simplified model of the type: *response ~ block + predictor + (1|plot ID)*. In these cases, values were only corrected for block and random effects. Graphical illustration of the trophic relationships was produced in R (package “bipartite,” Dormann, Gruber, & Fründ, 2008). For an analysis of species co‐occurrences, see supplement. Significant results are reported as mean ± SE derived from the final step‐2 model.

## 3 RESULTS

### 3.1 Description of the insect community in flower heads

**Figure 1:**
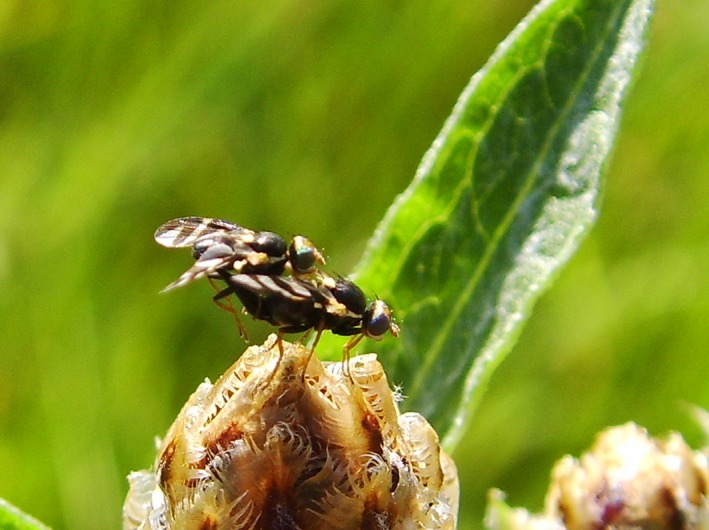
*Urophora quadrifasciata* on brown knapweed (*Centaurea jacea*). Picture: Sebastian König

**Figure 2:**
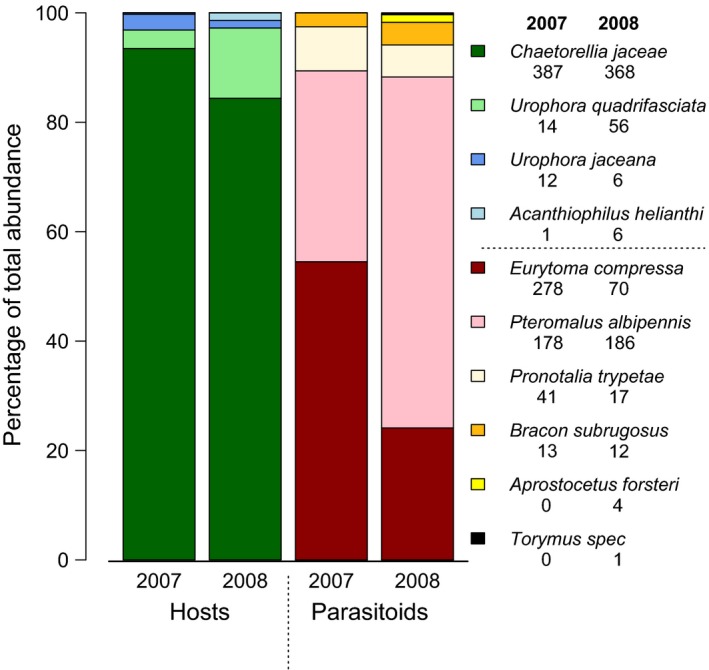
Composition of the host and parasitoid complex found in *Centaurea jacea* flower heads. Insect imagines abundances in 2007 and 2008 are listed below the species names in the legend on the right. Combined data from transplants (1–8 species plots) and potted plants (1–16 species plots)

We found 855 tephritid individuals in total and identified four tephritid species attacking *C. jacea*. The stenophagous *Chaetorellia jaceae* (Robineau‐Desvoidy, 1830) accounted for 93.5% and 84.4% of individuals in 2007 and 2008, respectively. *Urophora jaceana* (Hering, 1935), a gall‐inducing stenophagous tephritid, made up between 1% and 3% of individuals, whereas *Urophora quadrifasciata* (Meigen, 1826) (Figure 1) had similarly low abundance in 2007, but was more common in 2008, with 12.8% of individuals. The euryphagous *Acanthiophilus helianthi* (Rossi, 1794) was very rare (<2% of individuals) in both years (Figure 2, Table S4).

We collected 510 parasitoids in 2007 and 290 in 2008. We identified imagines of five endoparasitic Hymenoptera species: four chalcid wasps (*Aprostocetus forsteri* (Walker, 1847), *Eurytoma compressa* (Fabricius, 1794), *Pronotalia trypetae* Gradwell, 1957, *Pteromalus albipennis* Walker, 1835), and one braconid (*Bracon subrugosus* Szepligeti, 1901). *E. compressa* and *P. albipennis* dominated the parasitoid community, together accounting for almost 90% of imagines collected in either year. However, their relative proportions varied: *E. compressa* represented 54.5% and 24.1% in 2007 and 2008, respectively, whereas *P. albipennis* was less numerous in 2007 (34.9%) than in 2008 (64.1%) (Figure 2, Table S4). The two gregarious parasitoid species (*A. forsteri* and *P. trypetae*) were rare and a single individual of the ectoparasitic chalcid wasp *Torymus* cf. *chloromerus* was found in the 2008 collection.

**Figure 3:**
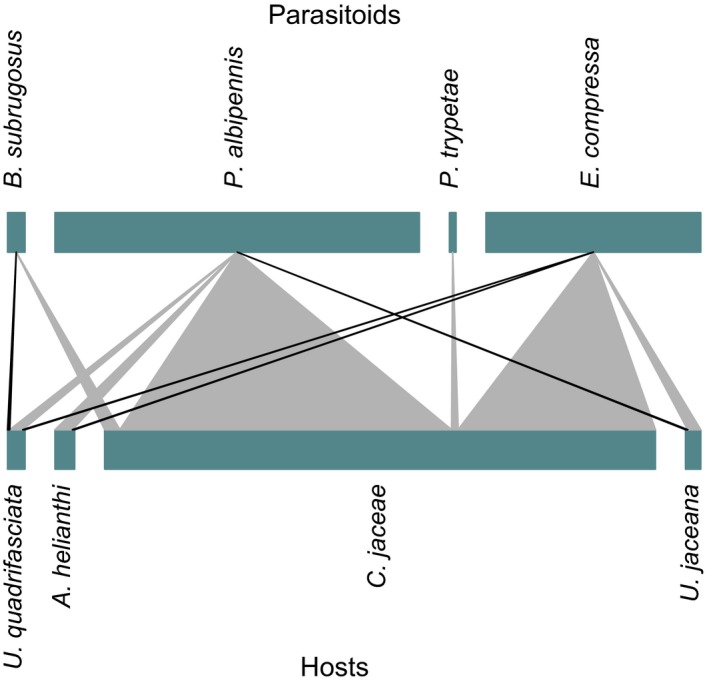
Host–parasitoid interaction web from combined data of 2007 and 2008. Species are represented by a rectangle, width of rectangle thereby reflecting their abundances. The lower rectangles list host species, and the upper line parasitoid species. The size of lines connecting the two trophic levels represents the interaction strength. Connections in black refer to interactions based on single observations

The two most abundant parasitoid species, *E. compressa* and *P. albipennis*, attacked all of the four tephritid host species and were most abundant on the commonest tephritid *Chaetorellia jaceae*. *E. compressa* was only once found parasitizing *A. helianthi* or *U. quadrifasciata* and *P. albipennis* was also only once found on *U. jaceana*. The braconid, *B. subrugosus*, mainly parasitized *C. jaceae* and only one interaction with *U. quadrifasciata* was recorded. The gregarious *P. trypetae* was only ever found in *C. jaceae*. The observed links between host and parasitoid species are summarized in Figure 3. Besides the tephritid hosts and their parasitoids, a number of other taxa associated with *Centaurea jacea* occurred in the samples (Chloropidae (Diptera, 67 imagines), Cecidomyiidae larvae (Diptera, uncounted), Cynipidae (Hymenoptera, 2 imagines), Lepidoptera larvae (8), *Cortinicara gibbosa* (Herbst 1793), (Coleoptera, 11 imagines)).

### 3.2 Responses of the second trophic level (Tephritidae) to plant diversity

**Figure 4:**
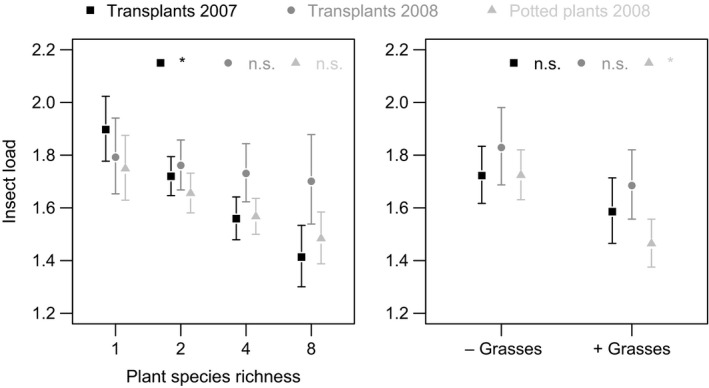
Influence of plant species richness and grass presence in experimental plant communities on *insect load* per flower head in the three datasets. Significance in final step‐2 models is abbreviated: * *p *≤* *.05, n.s. *p* > .05. Means ± SE derived from final step‐2 or simplified models (see Methods)

Only plant functional group presence affected the herbivorous Tephritidae in the analysis of potted plants. *Insect load* (i.e., the number of infestations per dissected flower head) was lower in the presence of grasses (1.5 ± 0.1 individuals) than in their absence (1.7 ± 0.1 individuals) (χ^2^ = 3.83, *p *=* *.050, Figure 4), both in the first and second step of the analysis (i.e., without and with covariates, Table 1). *Tephritid infestation rate* (i.e., the proportion of dissected flower heads that were infested) was not affected by any variables in the potted plants. Plant diversity and functional composition have therefore few direct effects on the herbivore community.

**Figure 5:**
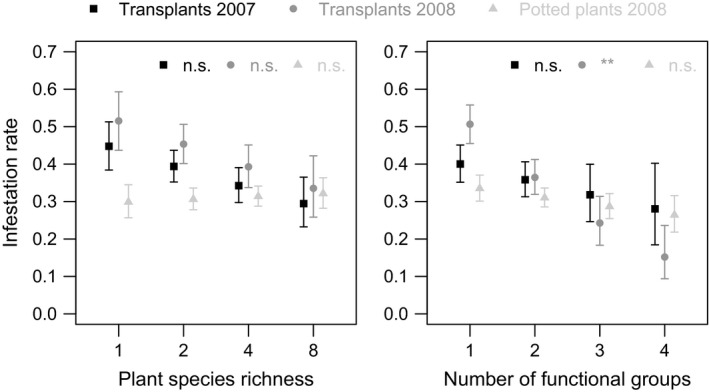
Influence of the number of plant species and functional groups present in experimental communities on tephritid *infestation rate* in the three datasets. Significance in final step‐2 models is abbreviated: ** *p *≤* *.005, n.s. *p *>* *.05. Means ± SE derived from final step‐2 or simplified models (see Methods)

Larger effects of plant diversity on herbivores were found when the transplant data from 2007 and 2008 (where both direct and indirect effects can be expected) were analyzed. In 2007, *insect load* decreased with increasing plant species richness from 1.9 ± 0.1 individuals per head in monocultures to 1.4 ± 0.1 individuals per head in 8‐species mixtures (χ^2^ = 6.40, *p *=* *.011, Figure 4); however, no significant effect was detected in 2008. The covariates did not have significant effects on *insect load* in either year (Table 1). *Tephritid infestation rate* responded more to functional diversity and decreased with increasing plant functional group richness by ca. 70% in 2008 (χ^2^ = 7.64, *p *=* *.006) but not in 2007 (Table 1, Figure 5). In the second step of the analysis, the *number of flower heads* was significant in both 2007 and 2008 (2007: χ^2^ = 14.73, *p *<* *.001; 2008: χ^2^ = 9.75, *p *=* *.002). *Tephritid infestation rate* increased with increasing resource density in both years (Table 1, Figure S2). These results suggest that plant species and functional group richness effects are stronger when both direct and indirect effects are operating.

### 3.3 Response of the third trophic level (parasitoids) to plant diversity

**Figure 6:**
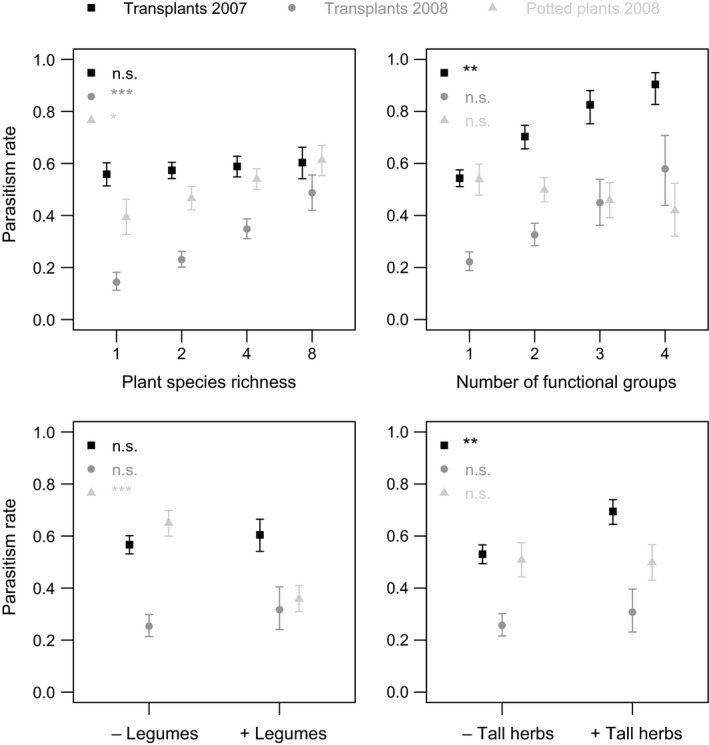
Influence of the number of plant species, functional groups, legume, and tall herb presence in experimental communities on *parasitism rate* in the three datasets. Significance in final step‐2 models is abbreviated: *** *p *≤* *.001, ** *p *≤* *.005, *p ≤* *.05, n.s. *p *>* *.05. Means ± SE derived from final step‐2 or simplified models (see Methods)

Plant species richness and functional group presence both affected parasitoid communities in the potted plants. In the first step of the analysis (without covariates), the presence of legumes reduced the *parasitism rate* (χ^2^ = 10.86, *p* = .001, Table 1, Figure 6: 35.8 ± 5.0% with legumes vs. 65.0 ± 5.0% without), and plant species richness had a marginally significant effect (χ^2^ = 3.73, *p* = .053). When covariates were included in the step‐2 model, plant species richness significantly increased *parasitism* (χ^2^ = 4.42, *p* = .036, Table 1, Figure 6). The *number of flower heads* also reduced *parasitism rate* in this model (χ^2^ = 4.19, *p* = .041, Table 1, Figure S3). We therefore find evidence for direct effects of plant species richness on parasitoids but not on herbivores.

**Figure 7:**
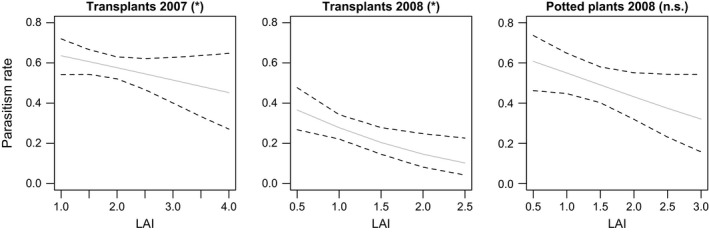
Influence of community “leaf area index” (LAI) on *parasitism rate* in the three datasets. Significance in final step‐2 models is abbreviated: * *p *≤* *.05, n.s. *p *>* *.05. Effects (grey line) and 95% confidence intervals (CI, dotted lines) derived from final step‐2 or simplified models (see Methods)

Plant species and functional group richness both affected *parasitism rate* in the transplants, where direct and indirect plant diversity effects could occur. Plant functional richness increased *parasitism rates* in 2007 from 54.2 ± 3.2% in plots with a single functional group to 90.4 ± 6.1% in plots with four functional groups (χ^2^ = 3.83, *p* = .050, Table 1, Figure 6). However, in 2008 plant species richness, rather than functional group richness, increased *parasitism rate* (from 14.4 ± 3.4% in monocultures to 48.8 ± 6.8% in 8‐species mixtures, χ^2^ = 8.63, *p* = .003, Table 1, Figure 6). The presence of tall herbs also increased parasitism rates by ca. 24% (χ^2^ = 4.62, *p* = .032, Figure 6). When adding covariates to the models in the second step of the analyses, an additional *LAI* effect was seen in transplants of both years (Table 1, Figure 7). Increasing *LAI* values in the plant communities reduced parasitism rates (2007: χ^2^ = 4.25, *p* = .039; 2008: χ^2^ = 5.88, *p* = .015). Including *LAI* in the model did not remove the other effects of the plant community (Table 1), suggesting that these are not mediated by changes in structural complexity. The lack of a significant *LAI* effect in potted plants (though pointing into the same direction, see Figure 7) may result from the generally slightly elevated position of potted plants that caused a proportion of them to be higher than the surrounding plant community. The covariates *Asteraceae presence*,* relative height*, and *tephritid infestation rate* never significantly affected parasitoids. Plant diversity effects on parasitoids were therefore found when direct effects alone and when both direct and indirect effects could operate. However, we were not able to explain the mechanisms driving these effects with our covariates.

## 4 DISCUSSION

### 4.1 Herbivores and parasitoids show opposing responses to plant diversity

Plant diversity had opposing effects on the herbivore and parasitoid communities of *Centaurea jacea* in a large grassland diversity experiment. Herbivore abundance (*infestation rate* and *insect load*) declined with increasing plant diversity, which agrees with the results of several previous studies (Balvanera et al., 2006; Haddad et al., 2009; Unsicker et al., 2006). However, these effects were variable and did not occur for all measures of the herbivore community or in all years. Herbivore populations are highly temporally variable (Solbreck & Sillén‐Tullberg, 1986; Walker, Hartley, & Jones, 2008) and plant diversity effects may only be detected in particular years. Plant diversity mostly tended to have negative (or in one case neutral) effects, so although there is variation in the strength and significance of effects, the direction is largely consistent. However, the variation in strength of the effect indicates that, although diversity may act in a similar way in different years, other drivers of herbivore abundance (such as climate or dispersal) may frequently mask the effects of plant diversity. In contrast, and in line with the *Enemies Hypothesis* (Root, 1973) and a range of more recent studies (Albrecht, Duelli, Schmid, & Muller, 2007; Bianchi et al., 2006; Haddad et al., 2009; Vanbergen, Hails, Watt, & Jones, 2006), parasitoid abundance increased with plant diversity. We found that both plant species richness and functional group richness were important components of diversity that affected the abundance of higher trophic levels. We compared responses on potted plants, where only direct effects of plant diversity, mediated by changes in resource density or apparency, can occur, with responses on transplants, where indirect effects, mediated by changing resource levels, could occur in addition to the direct effects. With this comparison, we were able to show that herbivore communities responded more strongly to indirect effects of plant diversity, perhaps mediated by changes in resource levels, whereas parasitoid communities responded more strongly to direct effects of plant diversity.

The two experimental variables *plant species richness* and *plant functional group richness* represent different aspects of plant diversity: *Functional group richness* effects suggest that plant species from the same functional group are redundant and have similar effects (i.e., similar plant characteristics), whereas effects of *plant species richness* imply that there is variation between species within functional groups (Roscher et al., 2004). It is not clear why the different measures of diversity should drive different measures of herbivore abundance and have effects in different years. However, these results suggest that both aspects of diversity may be important under certain conditions and that they may have similar effects (likely on plant quality and resource availability). *Plant species* and *functional group richness* also both reduced *C. jacea* performance traits (Nitschke et al., 2010), further supporting this idea.

### 4.2 Plant diversity indirectly affects the herbivorous Tephritidae

Plant diversity effects on Tephritidae were few and were only detected in the transplants where both direct and indirect effects of plant diversity can occur. The fact that no such effect was observed in potted plants (direct effects only) implies that these effects were largely mediated by changes in plant quality or performance traits along the plant diversity gradient. Accordingly, we found little evidence that apparency or the availability of alternative hosts in the plant community affected the herbivores. This may be because the herbivores (dominated by the monophagous *Chaetorellia jaceae*) are very efficient at finding their hosts and can locate them regardless of their surroundings. This is supported by the lack of an effect of structural complexity. We hypothesized that increasing structural complexity in the communities would impair the tephritids' host finding abilities, but our proxy for complexity (*LAI*) was never retained in the final herbivore models. We did find that grass presence reduced tephritid abundance, which might be explained by associational resistance effects (Barbosa et al., 2009); however, there is no evidence that this can explain the effect of plant diversity on herbivore abundance. Instead, variation in host plant performance and quality seem more likely to explain the plant diversity effects. Transplant performance (i.e., biomass, the number of flower heads) declines with increasing plant diversity (Nitschke et al., 2010) and this is likely to result in a decrease in food quality for the herbivores. In agreement with this idea, an increase in the number of flower heads did increase infestation rates in the transplants. This indicates that the Tephritidae are resource limited, which agrees with findings by Dempster et al. (1995). However, contrary to our expectations, resource density per host plant did not explain the effect of plant functional group richness on the herbivore community (i.e., functional richness remained significant when fitted alongside the number of flower heads). This suggests that other host plant characteristics must account for the negative effect of functional diversity.

In addition to host plant size, nutritional quality is also likely to affect herbivore communities. Nutritional quality is expected to decline with increasing plant diversity as a result of increased light competition and/or increased nutrient use efficiency in diverse plant communities, and thus to negatively affect herbivore abundance (Abbas et al., 2013; but see Ebeling et al., 2014). The opposite pattern could occur if shading reduces plant chemical defenses and increases specific leaf area and hence palatability (Crone & Jones, 1999; Guerra, Becerra, & Gianoli, 2010; Mraja, Unsicker, Reichelt, Gershenzon, & Roscher, 2011). However, the negative response of tephritid species to diversity suggests a decline in plant quality in this case. A decline in plant quality could affect oviposition, as many tephritid species can assess host plant quality and adjust clutch size in response (Burkhardt & Zwölfer, 2002; Freese & Zwölfer, 1996; Pittara & Katsoyannos, 1992; Rieder, Evans, & Durham, 2001). For instance, Burkhardt and Zwölfer (2002) found that ovipositing females of the gall forming *U. jaceana* preferred high‐quality plants and flower heads which resulted in increased larval growth and fecundity. The production of a gall is costly and time‐consuming, which means that there is likely to be a strong advantage for *U. jaceana* in assessing host plant quality and not investing in poor quality plants. Other monophagous species like *Chaetorellia jaceae*, the most abundant species in our study, may also have evolved mechanisms to assess host plant quality. We might expect very different patterns for more generalist herbivores that could benefit from diet mixing in diverse plant communities (Pfisterer, Diemer, & Schmid, 2003). These negative indirect effects of plant diversity on herbivore communities, mediated by changes in host plant quality, have been largely overlooked but our results suggest that they may be important, particularly for monophagous species.

### 4.3 Plant diversity directly affects parasitoid communities

In accordance with predictions from the *Enemies Hypothesis* (Root, 1973), we found that parasitism rates increased with increasing plant functional group or species richness. This agrees with an observational study, where plant diversity was affected by grazing intensity, which found that the parasitism rate by *Pteromalus elevatus* on the tephritid *Tephritis conura* increased with plant species richness (Vanbergen et al., 2006). Our study allows us to look at the mechanisms driving these effects in more detail. As the diversity effect in potted plants cannot be attributed to changes in host plant performance, it must be caused by changes in the plant community, which suggests that direct effects of diversity are the most important. One of the main direct effects of plant diversity may be to increase the availability of floral resources, which is expected to benefit parasitoids through increased nectar provision (e.g., Araj et al., 2008; Lavandero et al., 2006). In our study system, flower availability increases with increasing plant species richness (Ebeling, Klein, Schumacher, Weisser, & Tscharntke, 2008), which means diverse communities would provide more resources for parasitoids. The covariate *Asteraceae presence* in the plant community did not affect parasitoid abundance, which suggests that the parasitoids may be quite generalist and able to feed on a range of flowering plants. This is supported by the fact that the presence of tall herbs in the community did significantly increase parasitism rates in transplants. In contrast, the presence of legumes decreased parasitism and this might be explained if legume presence results in a smaller relative abundance of other herbs. This would reduce parasitoid abundance if parasitoids do not feed on legume flowers and prefer open flowers such as those of tall herbs in the Asteraceae, Apiaceae, and Dipsacaceae families. Indeed, studies on the attractiveness of various plant species to nectar feeding parasitoids suggest that Chalcidoidea preferentially visit open and easily accessible inflorescences such as those of Apiaceae and Asteraceae (Sivinski, Wahl, Holler, Al Dobai, & Sivinski, 2011) and avoid legumes (Jervis, Kidd, Fitton, Huddleston, & Dawah, 1993). Measuring the cover of different flower types in the plots would be needed to explicitly test this idea. Moreover, extra‐floral nectaries (EFNs) in plants may serve as an alternative nectar source for parasitoids, and if diverse communities contain more plants with EFNs then this could explain our results. Only 3 of our 60 species have EFNs according to the “World List of Plants with EFNs” (Weber, Porturas, & Keeler, 2015). However, the list is not fully comprehensive and only contains those species currently reported to have EFNs. Further measures of EFNs on the field site would be needed to test for such an effect. In addition to these potential effects of floral resource availability, parasitism rate may be affected by host resource availability. *Tephritid infestation rate* and *insect load* both decreased with increasing plant diversity (functional group or species richness), which means the increase in parasitism could also be driven by decreasing host density. This response was reported in a study on trap nesting bees on the same field site (Ebeling et al., 2012). However, such an effect seems less likely as an explanation of our results because we found that *tephritid infestation rate* did not affect parasitism rate. This is in accordance with results from a study by Walker et al. (2008), which implies that host density was either not a main driver of parasitism rates or that parasitoids cannot easily determine host density. The latter notion is supported by a reduction in *parasitism rates* when the *number of flower heads* was high in potted plants. This might indicate decreasing parasitoid efficiency with an increasing number of potential host locations, that is, a “dilution effect.” The parasitoids that we found can attack herbivores associated with some other Asteraceae species found on our field site, with almost no other associations recorded (Noyes, 2016). However, the variable *Asteraceae presence* did not affect parasitoid parameters, which implies that parasitoids were not using alternative hosts within the high diversity communities. We detected another direct community effect on parasitoid abundance: our proxy for structural complexity (*LAI*) significantly reduced parasitism rate suggesting that host finding was impaired by increasing complexity of the vegetation. Although LAI is positively related to plant diversity (Spehn et al., 2000), it has opposing effects on parasitoid abundance. This implies that, although an increase in structural complexity might reduce the ability of parasitoids to find their hosts in diverse plant communities, other benefits of plant diversity are sufficient to override this effects.

Our results suggest that diverse plant communities harbor a more efficient parasitoid community, likely because of a greater provision of floral resources.

### 4.4 General conclusion

Our study in a model tri‐trophic system—*Centaurea jacea*, Tephritidae, parasitoids—suggests opposing responses of herbivores and parasitoids to plant diversity with clearer effects seen in parasitoids than in herbivores. The negative herbivore response seems to be mostly driven by changes in host plant quality. This suggests that some of the negative effects of plant diversity on herbivore abundance found in previous studies could be explained by these more indirect effects on plant quality. Future studies should therefore consider controlling for changes in quality and potted plants placed into communities are a useful way to do this. In contrast to the herbivores, parasitoid abundance increased with diversity. This seems to be partly driven by increased resource availability (i.e., nectar and pollen) although direct measures of the resources available to parasitoids would be needed to confirm this. The increase in parasitism rate (and decrease in herbivory) also argue for the value of diverse plant communities in providing more efficient pest control. Our results show that plant diversity is a key driver of the abundance of higher trophic levels and that a wide variety of mechanisms can operate to explain these effects.

## CONFLICT OF INTERESTS

None declared.

## DATA ACCESSIBILITY

Data will be made publicly available via the data publisher “Pangaea” (https://pangaea.de).
