# Supplementary material for: Plant diversity has contrasting effects on herbivore and parasitoid abundance in Centaurea jacea flower heads
Source: Ecol Evol. 2017 Oct 5;7(22):9319–32. doi: 10.1002/ece3.3142 (PMC5696411; doi:10.1002/ece3.3142)
Supplement: Supplementary file 1 [file ECE3-7-9319-s001.docx]

**Supplementary Information**

**Contents**

Methodological details – insect identification and trophic relationships 1

Supplementary tables and figures 2

Co-occurrence analyses 8

References 11

***Methodological details - Insect identification and trophic relationships***

As only a very small number of potential hosts other than Tephritidae were found when dissecting flower heads (i.e. a few Lepidoptera and Hymenoptera (*Isocolus* spec.) larvae) and as evidence of parasitism of these alternative hosts was lacking, all parasitoids detected were considered to have originated from tephritid hosts. This was consistent with the host records known for the parasitoid species encountered.

All tephritid hosts were identified using the key by Merz (1994). Parasitoids were identified as follows: *Pteromalus* individuals were verified with Graham (1967), a subsample of *Eurytoma* individuals was verified by an external specialist (Gérard Delvare, CIRAD, F), *Pronotalia* and *Aprostocetus* individuals were identified with Graham (1987, 1991), and *Bracon* individuals were identified by an external specialist (Kees van Achterberg, Leiden, NL). Taxonomy follows Fauna Europaea (de Jong, 2011).

In order to determine trophic interactions from the dissection data the following rules were employed: (i) Parasitoid attribution to a host species was evident in cases where parasitoids were found in tephritid pupae or galls. (ii) In all solitary parasitoids detected, individuals were considered to have emerged from a single host. Consequently, when only one tephritid species was present in a flower head, and the number of emerged solitary endoparasitoids matched the number of empty tephritid pupae, the respective parasitoids were assigned to that host. In a few cases, we detected gregarious species (*Pronotalia trypetae* Gradwell, 1957 and *Aprostocetus forsteri* (Walker, 1847)), for which assignment to the host species was unambiguous only where a clutch was found together with a single, empty host pupa. (iii) When there were several possible host species, individual parasitoids were not assigned to any host. (iv) When there were parasitoids but no obvious sign of the host, no assignment was undertaken either. Data from cases (iii) and (iv) were excluded from analysis of particular host-parasitoid species relationships (see co-occurrence analysis below). Graphical illustration of the trophic relationships was produced in R (package “bipartite”, Dormann et al., 2008).

***Supplementary Tables and Figures***

**Table S1:** Functional group composition of experimental mixtures in the Jena Experiment at set up in 2002. Table taken from Roscher et al., 2004 (there: Table 3, page 113).

**
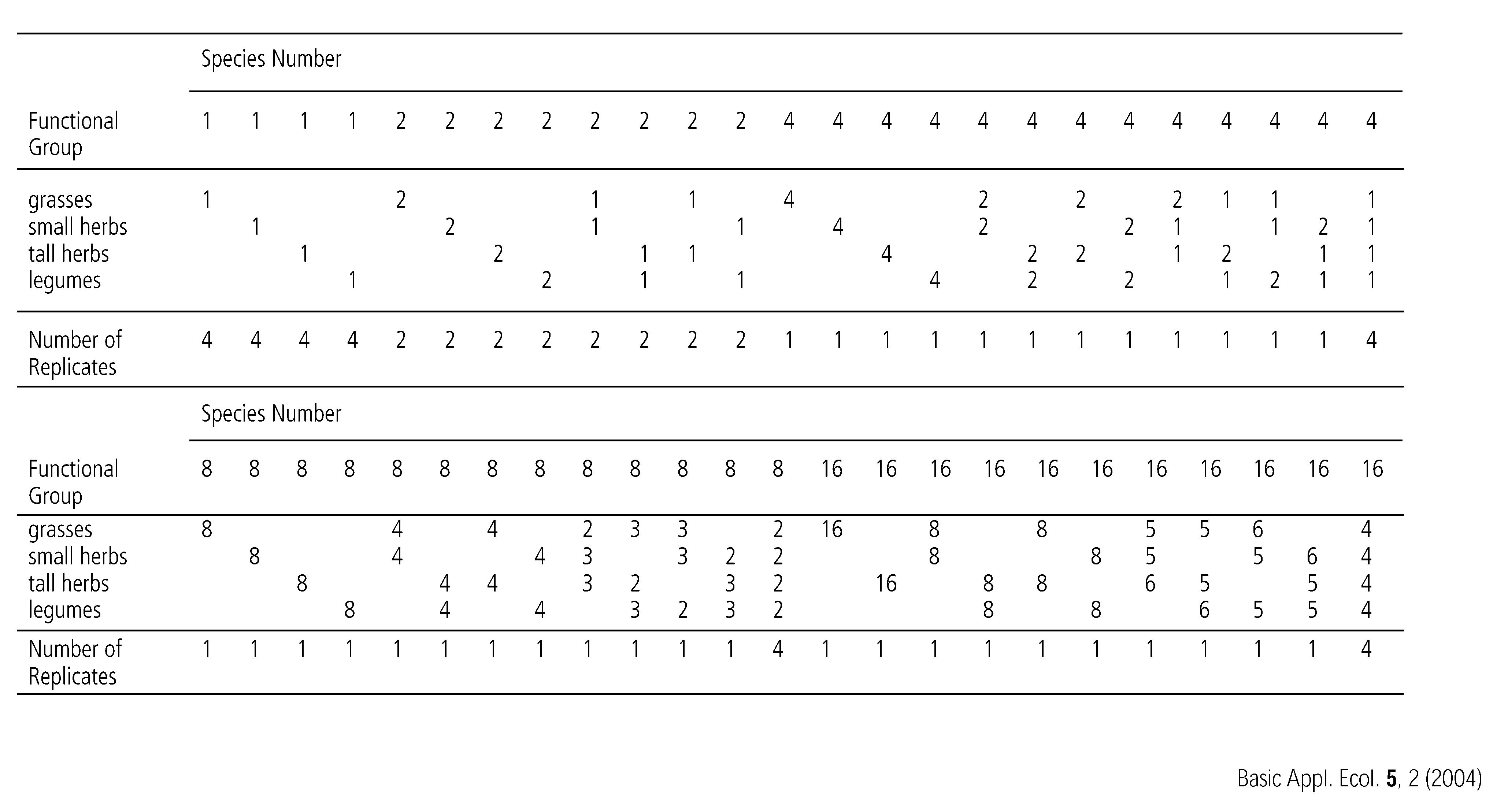
**

**Table S2:** Experimental time frame indicating field site mowing, removal of flower heads, placement in experimental plots and harvest of transplants and potted plants in both years.

| **Week no.** | **25** | **26** | **27** | **28** | **29** | **30** | **31** | **32** | **33** | **34** | **35** | **36** |
| --- | --- | --- | --- | --- | --- | --- | --- | --- | --- | --- | --- | --- |
| Transplants 2007 | *First mowing*  *(all plants cut)* |  |  |  |  |  |  |  |  |  | *Harvest* | *Second mowing* |
| Transplants 2008 |  |  |  |  |  |  |  |  |  |  |  |  |
| Potted Plants 2008 |  |  |  | *Heads*  *cut off,*  *placed*  *in plots* |  |  |  |  |  |  |  |  |

**Table S3:** Distribution of flower head bearing transplants (and pots) across the experimental plant communities. The numbers of plants (*plants*) and experimental plots (*plots*) are specified for the different experimental factors implemented in the experimental field design (Div=plant species richness, NRFG=functional group richness, FG=plant communities in which the particular functional group is present (+) or absent (-); Leg=legumes, Grass=grasses, Sherb=small herbs, Therb=tall herbs).

| Year | Factor | **Transplants/ Pots** | | | | | | | |
| --- | --- | --- | --- | --- | --- | --- | --- | --- | --- |
|  | **Div** | **1** | **2** | | **4** | **8** | **16** | | **60** |
| 2007 | *plants* | 34 | 40 | | 22 | 26 | 13 | | 0 |
|  | *plots* | 11 | 12 | | 11 | 10 | 7 | | 0 |
| 2008 | *plants* | 29/36 | 30/41 | | 20/41 | 25/46 | 2/39 | | 0/11 |
|  | *plots* | 11/14 | 12/16 | | 9/16 | 10/16 | 2/14 | | 0/4 |
|  | **NRFG** | **1** | | **2** | | **3** | | **4** | |
| 2007 | *plants* | 81 | | 31 | | 10 | | 13 | |
|  | *plots* | 24 | | 13 | | 7 | | 7 | |
| 2008 | *plants* | 62/82 | | 29/55 | | 10/33 | | 5/44 | |
|  | *plots* | 25/32 | | 11/20 | | 5/12 | | 3/16 | |
|  | **FG** | **Leg +/-** | | **Grass +/-** | | **Sherb +/-** | | **Therb +/-** | |
| 2007 | *plants* | 34/101 | | 66/69 | | 66/69 | | 59/76 | |
|  | *plots* | 19/32 | | 29/22 | | 26/25 | | 25/26 | |
| 2008 | *plants* | 26/80 116/98 | | 55/51 117/97 | | 54/52 117/97 | | 35/71 117/97 | |
|  | *plots* | 14/30 43/37 | | 21/23 43/37 | | 21/23 42/38 | | 18/26 44/36 | |

**Table S4:** Numbers of imagine host and parasitoid individuals emerging/dissected from *Centaurea jacea* transplants and potted plants in 2007 and 2008 (transplants from: 1-8; potted plants from: 1-16 plant species communities).

|  | |  | **2007**  *transplants* | **2008**  *transplants* | **2008**  *potted plants* |
| --- | --- | --- | --- | --- | --- |
| **Number of plants** | | | 204 | 191 | 246 |
| **Number of heads on plants** | | | 3684 | 2650 | 2441 |
| **Insect numbers** *emerged* | # Hosts  (infestation rate) | | 348 (22%) |  |  |
|  | # Parasitoids (parasitism rate) | | 463 (57%) |  |  |
| **Number of heads dissected**  (proportion of total) | | | 644 (17,5%) | 530 (20,0%) | 1078 (44,2%) |
| **Insect numbers** *dissected* | # Hosts  (infestation rate) | | 71 (18,3%) | 245 (65,5%) | 191 (35,2%) |
|  | # Parasitoids (parasitism rate) | | 47 (39,8%) | 102 (29,4%) | 188 (49,6%) |


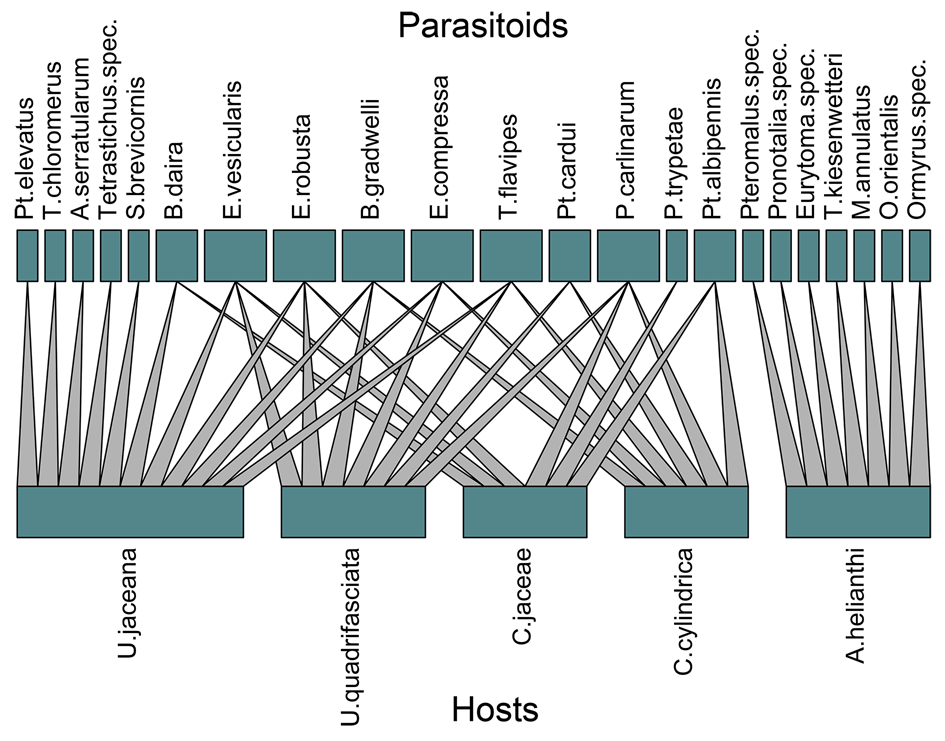


**Figure S1:** Potential interaction web for the *Centaurea jacea* flower head insect community (data derived from the Universal Chalcidoidea Database, Noyes, 2013). The number of potential chalcid wasp parasitoid species almost complies with the number of parasitoids listed by Hoffmeister and Vidal (1994) in a literature compilation, suggesting the predominance of chalcid wasp parasitoids in *Centaurea jacea*.


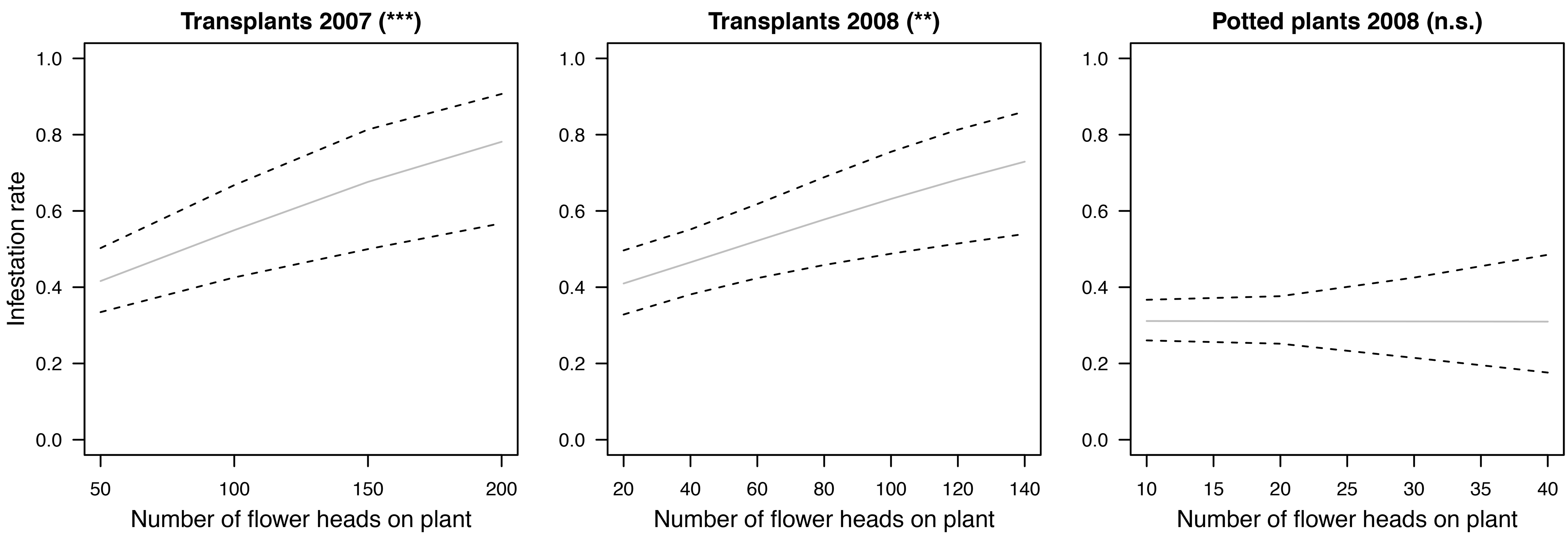


**Figure S2**: Positive effect (grey line) and 95% confidence intervals (CI, dotted lines) of flower head numbers on tephritid infestation rate in *Centaurea jacea* plants. Significances in final step-2 models: *** *P* ≤ 0.001, ** *P* ≤ 0.01, n.s. *P* > 0.05. Effects and CIs derived from final step-2 or simplified models (see Methods).


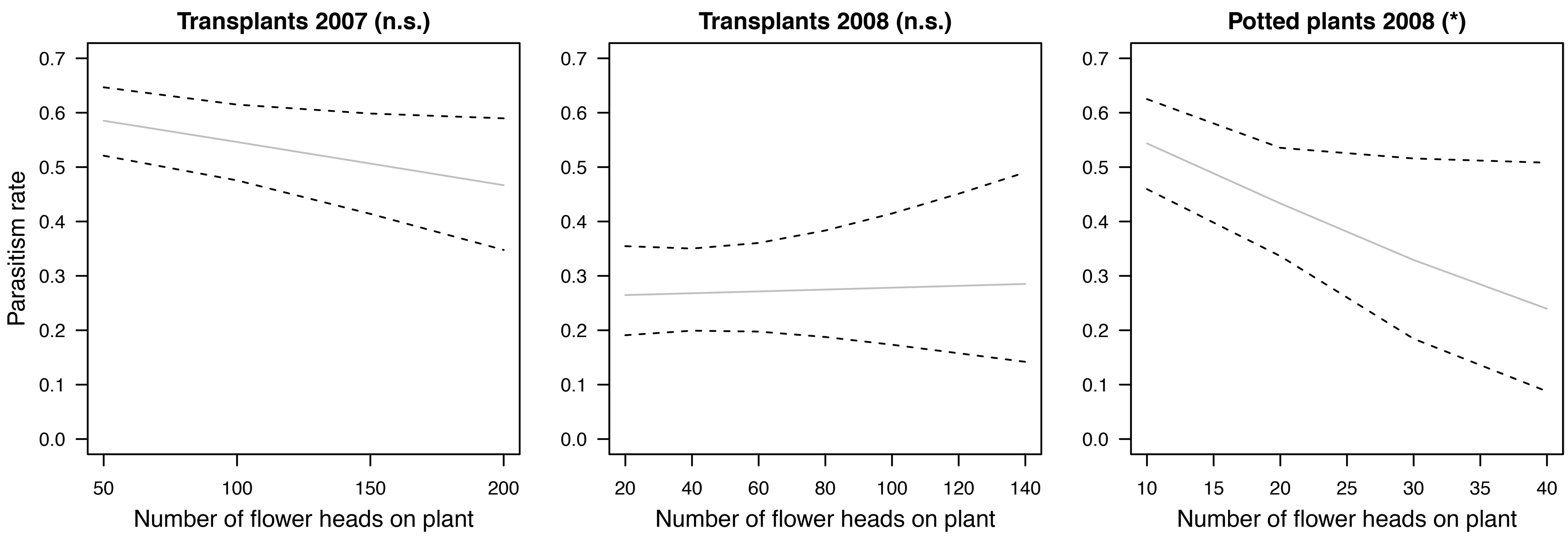


**Figure S3**: Negative effect (grey line) and 95% confidence intervals (CI, dotted lines) of flower head numbers on parasitism rates in *Centaurea jacea* potted plants for plant diversity of one to eight species. Significances in final step-2 models: * *P* ≤ 0.05, n.s. *P* > 0.05. Effects and CIs derived from final step-2 or simplified models (see Methods).


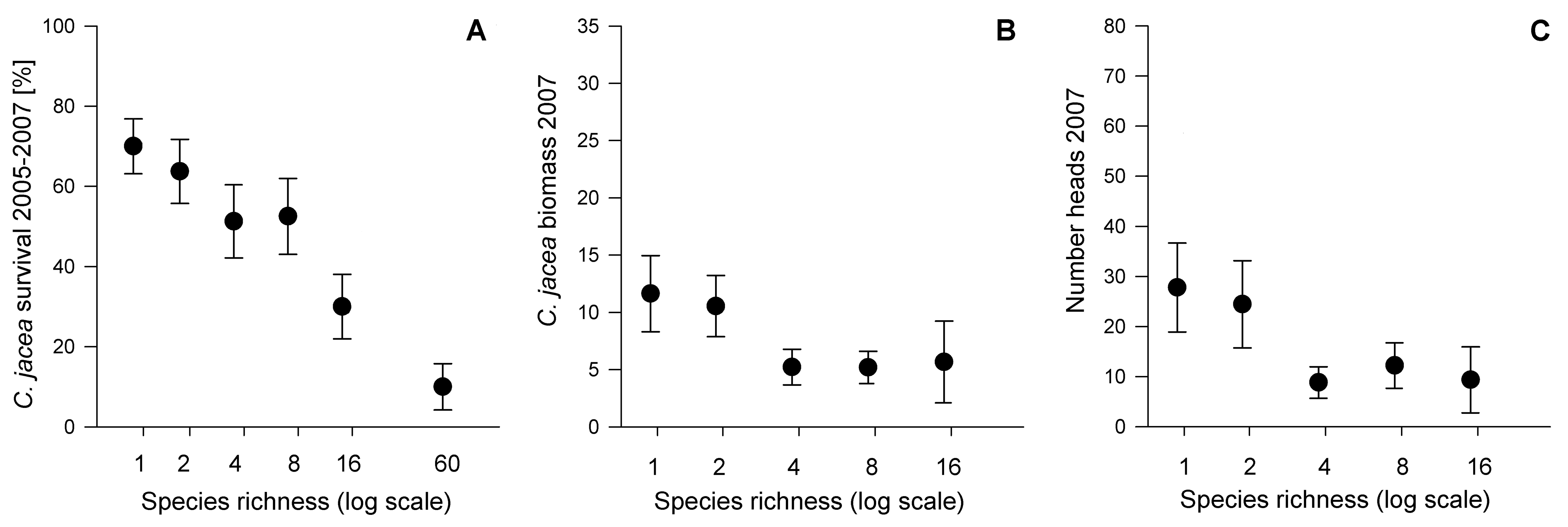


**Figure S4**: Effects of plant species richness on (A) survival, (B) biomass and (C) flower head production in *Centaurea jacea* transplants sampled in 2007. Graphs show plot mean values ± SE. Modified after Nitschke et al., 2010.

***Co-occurrence analyses***

To investigate patterns of species co-occurrence in *Centaurea jacea* flower heads, we analysed three types of contingency tables with host and parasitoid data from 2008. Dissection data on trophic relationships from transplants and potted plants were pooled. The first approach (*interspecific co-occurrence*) tested hosts and parasitoids separately, investigating if particular species, of the same trophic level, occurred with heterospecifics less often than expected. We used data from flower heads in which two host or parasitoid individuals were found and the contingency table listed how often each species occurred with a conspecific or with a heterospecific. In the second approach (*intratrophic co-occurrence*) we tested whether particular host or parasitoid species avoided development with other individuals of the same trophic level, listing for each species in how many cases it occurred alone, or with one, two, three, or more other individuals. The third approach (*parasitoid specificity*) tested, whether a preference of parasitoid species for host species could be detected. Here, the number of interactions for each host × parasitoid species combination was listed. In all approaches ‘Fisher’s Exact Test’ was used for analysis of contingency tables, due to low numbers of observations for several cells. Association plots were used to determine the direction of effects. Analysis was conducted in R.

***Results: Co-occurrence analyses***

A total of 1608 flower heads (transplants and potted plants) from the 2008 collection was dissected (suppl. Table S4), and out of these 601 were infested with Tephritidae. In 52% of these cases, only one host individual was present in a flower head, in 27% there were two, in 13% there were three, and in 8% there were four or more individuals per flower head.

Our analysis of *interspecific co-occurrence* indicated that when there were two host individuals in a flower head, individuals belonged more often than expected to the same species than to different ones (*P* < 0.001). For parasitoids, there was no deviance from the expected distribution (*P* = 0.790).

The approach on *intratrophic co-occurrence* indicated that the most abundant host species *C. jaceae* occurred more often than expected singly in a flower head, while all other host species occurred more often than expected with one or more other individuals in a flower head (*P* < 0.001). There was no significant departure from the expected distribution in parasitoids (*P* = 0.662).

The approach on *parasitoid specificity* indicated no departure of the observed number of specific host × parasitoid interactions from the expected distribution, given the respective numbers of individuals of each species (*P* = 0.712).

***Discussion: Species-specific attack rates***

Among all dissected flower heads which showed tephritid infestation, about half were infested with only one individual. Analysis of flower heads with two individuals (27%) hints at stronger interspecific than intraspecific competition in tephritids, as co-occurrence with heterospecifics was less likely. This is surprising as the four tephritids show different feeding strategies, which should allow for resource partitioning within a single flower head (Zwölfer, 1988), while similar strategies of resource use should lead to higher competition, especially in small flower heads and in non-galling species. Although we did not record flight periods of the different tephritid species, temporal separation of activity peaks may have promoted the observed pattern as the probability of encountering a conspecific would be larger than encountering a hetero­specific. For *Acanthiophilus helianthi*, an earlier activity peak is likely as in this species mostly empty pupae were detected. However, in the analysis expected distribution is based on the relative proportion of species found. Therefore, deviance from that distribution cannot wholly be explained by different activity peaks.

In contrast to the two gall-forming species *Urophora quadrifasciata* and *U. jaceana*, the free-living *Chaetorellia jaceae* appeared to avoid multiple infestations of flower heads and occurred singly more often than expected. This pattern matches the feeding strategies of the different species. One third of all gall forming species in flower heads lays eggs in clutches of two and more (Headrick and Goeden, 1998) and studies on *U. cardui* even suggest benefits from larger clutch sizes as the risk of parasitism is decreased and resource supply increased with increasing clutch size (Freese and Zwölfer, 1996, Zwölfer and Arnold-Rinehart, 1993). *C. jaceae* on the contrary feeds destructively within the flower head and consumes large proportions of available tissue (N.N. pers. obs.; Dempster et al., 1995) thereby strongly limiting seed set possibility. Several mechanisms are known by which Tephritidae are able to avoid competition for resources within a fruit or flower head, including the use of oviposition deterring pheromones (Roitberg and Prokopy, 1987, Straw, 1989), the ability to assess resource quality prior to oviposition (Freese and Zwölfer, 1996), and behavioural adjustments, e.g. in emigration tendency (Romstöck-Völkl, 1990).

We did not detect any preferences in parasitoid occurrence which shows that at the flower head level, parasitoids did not distinguish between heads that already contained a parasitoid or not, nor between the parasitoid species already present. For parasitoids, however, the relevant resource level is the particular tephritid host individual because in solitary species, commonly only one larva per host individual develops to the adult stage. This high mortality risk of multiple parasitoid oviposition may be lowered by patch or host markings (Hoffmeister and Roitberg, 1997), but the actual number of ovipositions per tephritid hosts could not be monitored with the approach taken.

Finally, no specific preferences of a particular parasitoid species for a particular tephritid species were detected and this observation is most likely due to the dominance of a single tephritid host species and the low host specificity of the two dominant parasitoid species.

The low individual numbers in tephritid species other than *C. jaceae* and in parasitoid species other than *E. compressa* and *P. albipennis* impair reliable statements concerning the rarer insect species detected, and the overall attack patterns are driven by these three most abundant species.

**Data Accessibility**

Data will be made publicly available via the data publisher ‘Pangaea’ (https://pangaea.de).

**References**

DE JONG, Y. S. D. M. 2011. *Fauna Europaea* [Online]. Available: http://www.faunaeur.org/ [Accessed].

DEMPSTER, J. P., ATKINSON, D. A. & CHEESMAN, O. D. 1995. The spatial population-dynamics of insects exploiting a patchy food resource - 1. Population extinctions and regulation. *Oecologia,* 104**,** 340-353.

DORMANN, C. F., GRUBER, B. & FRÜND, J. 2008. Introducing the bipartite package: Analysing ecological networks. *R-News,* 8**,** 8-11.

FREESE, G. & ZWÖLFER, H. 1996. The problem of optimal clutch size in a tritrophic system: The oviposition strategy of the thistle gallfly *Urophora cardui* (Diptera, Tephritidae). *Oecologia,* 108**,** 293-302.

GRAHAM, M. W. R. D. V. 1967. Pteromalidae of north western Europe (Hymenoptera: Chalcidoidea). *Bulletin of the British Museum (Natural History) Entomology, Supplement,* 16**,** 908.

GRAHAM, M. W. R. D. V. 1987. A reclassification of the European Tetrastichinae (Hymenoptera: Eulophidae), with a revision of certain genera. *Bulletin of the British Museum (Natural History) Entomology,* 55**,** 392.

GRAHAM, M. W. R. D. V. 1991. A reclassification of the European Tetrastichinae (Hymenoptera: Eulophidae): revision of the remaining genera. *Memoirs of the American Entomological Institute,* 49**,** 322.

HEADRICK, D. H. & GOEDEN, R. D. 1998. The biology of nonfrugivorous tephritid fruit flies. *Annual Review of Entomology,* 43**,** 217-241.

HOFFMEISTER, T. S. & ROITBERG, B. D. 1997. To mark the host or the patch: Decisions of a parasitoid searching for concealed host larvae. *Evolutionary Ecology,* 11**,** 145-168.

HOFFMEISTER, T. S. & VIDAL, S. 1994. The diversity of fruit fly (Diptera: Tephritidae) parasitoids. *In:* HAWKINS, B. A. & SHEEHAN, W. (eds.) *Parasitoid Community Ecology.* Oxford: Oxford University Press.

MERZ, B. 1994. *Tephritidae (Diptera)*, Schweizerische Entomologische Gesellschaft.

NOYES, J. S. 2013. *Universal Chalcidoidea Database* [Online]. The Natural History Museum, London. http://www.nhm.ac.uk/entomology/chalcidoids/index.html. [access 13.09.2013]

ROITBERG, B. D. & PROKOPY, R. J. 1987. Insects that mark host plants. *Bioscience,* 37**,** 400-406.

ROMSTÖCK-VÖLKL, M. 1990. Population-dynamics of *Tephritis conura*, Loew (Diptera, Tephritidae) - Determinants of density from three trophic levels. *Journal of Animal Ecology,* 59**,** 251-268.

ROSCHER, C., SCHUMACHER, J., BAADE, J., WILCKE, W., GLEIXNER, G., WEISSER, W. W., SCHMID, B. & SCHULZE, E. D. 2004. The role of biodiversity for element cycling and trophic interactions: an experimental approach in a grassland community. *Basic and Applied Ecology,* 5**,** 107-121.STRAW, N. A. 1989. Evidence for an oviposition-deterrring pheromone in *Tephritis bardanae* (Schrank) (Diptera, Tephritidae). *Oecologia,* 78**,** 121-130.

ZWÖLFER, H. 1988. Evolutionary and ecological relationships of the insect fauna of thistles. *Annual Review of Entomology,* 33**,** 103-122.

ZWÖLFER, H. & ARNOLD-RINEHART, J. 1993. The Evolution of Interactions and Diversity in Plant-Insect Systems: The *Urophora-Eurytoma* Food Web in Galls on Palaearctic Cardueae. *In:* SCHULZE, E. D. & MOONEY, H. A. (eds.) *Biodiversity and Ecosystem Function.* Heidelberg: Springer.
